# The prevalence and impact of sarcopenia in older cardiac patients undergoing inpatient cardiac rehabilitation – results from a prospective, observational cohort pre-study

**DOI:** 10.1186/s12877-024-04694-y

**Published:** 2024-01-24

**Authors:** Carolin Steinmetz, Laura Krause, Samra Sulejmanovic, Sabrina Kaumkötter, Thomas Mengden, Clemens Grefe, Ernst Knoglinger, Nils Reiss, Klara Brixius, Birna Bjarnason-Wehrens, Thomas Schmidt, Stephan von Haehling, Monika Sadlonova, Christine A. F. von Arnim, Stephanie Heinemann

**Affiliations:** 1 Department of Geriatrics, University Medical Center Goettingen, Robert-Koch-Str. 40, 37075 Goettingen, Germany; 2 Institute for Sports Science University of Hildesheim, Universitätsplatz 1, 31141 Hildesheim, Germany; 3 Schüchtermann-Schiller’sche Clinic, Ulmenallee 11, 49214, Bad Rothenfelde, Germany; 4 Department of Rehabilitation, Kerckhoff Heart Center, Ludwigstr. 41, 61231 Bad Nauheim, Germany; 5 Clinic and Rehabilitation Center Lippoldsberg, Birkenallee 1, 34399, Wesertal, Germany; 6 Kirchberg Clinic, Bad Lauterberg, Kirchberg 7-11, 37431 Bad Lauterberg, Germany; 7 Institute of Cardiology and Sports Medicine, Department of Molecular and Cellular Sports Medicine, German Sport University, Am Müngersdorfer Sportpark 6, 50933 Cologne, Germany; 8 Institute of Cardiology and Sports Medicine, Department Preventive and Rehabilitative Sport and Exercise Medicine, German Sport University, Am Müngersdorfer Sportpark 6, 50933 Cologne, Germany; 9 Department of Cardiology and Pneumology, University Medical Center Goettingen, Robert-Koch-Str. 40, 37075 Goettingen, Germany; 10 Department of Psychosomatic Medicine and Psychotherapy, University of Goettingen Medical Center, Robert-Koch-Str. 40, 37075 Goettingen, Germany; 11 Department of Cardiovascular and Thoracic Surgery, University of Goettingen Medical Center, Robert-Koch-Str. 40, 37075 Goettingen, Germany; 12 German Center for Cardiovascular Research (DZHK), Partner Site Goettingen, Robert-Koch-Str. 42a, 37075 Goettingen, Germany

**Keywords:** Cardiac surgery, Valve intervention, Sarcopenia, Cardiac rehabilitation

## Abstract

**Background:**

The prevalence of sarcopenia and its impact in older patients undergoing inpatient cardiac rehabilitation (iCR) after cardiac procedure has been insufficiently studied. The main aim of this study was to evaluate the prevalence of sarcopenia and quantify the functional capacity of older sarcopenic and non-sarcopenic patients participating in iCR.

**Methods:**

Prospective, observational cohort study within the framework of the ongoing multicenter prehabilitation study “PRECOVERY”. A sample of 122 patients ≥75 years undergoing iCR after cardiac procedure were recruited in four German iCR facilities and followed up 3 months later by telephone. At iCR (baseline), the Strength, Assistance with walking, Rise from a chair, Climb stairs and Falls (SARC-F) questionnaire was used to identify sarcopenic patients. In addition, Katz-Index, Clinical Frailty Scale (CFS), handgrip strength (HGS), Short Physical Performance Battery (SPPB) and 6-minute walk distance (6MWD) measured functional capacity and frailty at baseline. Outcomes were prevalence of sarcopenia and the correlation of sarcopenia to functional capacity and frailty at baseline as well as the SARC-F score at follow-up. The Wilcoxon test was applied for pre-post-test analysis. Correlation between sarcopenia and 6MWD, SPPB score and HGS was tested with the eta coefficient with one-way ANOVA.

**Results:**

Complete assessments were collected from 101 patients (79.9 ± 4.0 years; 63% male). At baseline, the mean SARC-F score was 2.7 ± 2.1; 35% with sarcopenia. Other baseline results were Katz-Index 5.7 ± 0.9, CFS 3.2 ± 1.4, HGS 24.9 ± 9.9 kg, SPPB score 7.5 ± 3.3 and 6MWD 288.8 ± 136.5 m. Compared to baseline, fewer patients were sarcopenic (23% versus 35%) at follow-up. In the subgroup of sarcopenic patients at baseline (*n* = 35), pre-post comparison resulted in a significant SARC-F improvement (*p* = 0.017). There was a significant correlation between sarcopenia measured by SARC-F and poor results in the assessments of functional capacity (*p* < 0.001; r > 0.546).

**Conclusions:**

The prevalence of sarcopenia in older patients at iCR after cardiac procedure is high (35%) and remains high at follow-up (23%). Sarcopenia screening is important since the diagnosis of sarcopenia in these patients correlates significantly with poor functional capacity. The results indicate that these patients may benefit from prehabilitation aimed at improving perioperative outcomes, increasing functional capacity and mitigating adverse effects.

**Trial registration:**

German Clinical Trials Register (DRKS; http://www.drks.de; DRKS00032256). Retrospectively registered on 13 July 2023.

**Supplementary Information:**

The online version contains supplementary material available at 10.1186/s12877-024-04694-y.

## Introduction

### Prevalence of sarcopenia

Patients with sarcopenia have higher risk of falls and fractures, physical limitations and poorer quality of life [1, 2]. In its most severe form, sarcopenia is associated with increased frailty, morbidity, and mortality [3]. Concretely, patients with sarcopenia have a 3.2 times increased risk of falls [4] and a 3.6 times higher mortality [5] than patients without sarcopenia. Sarcopenia can be also associated with an increase in visceral fat - so called sarcopenic obesity - which favors chronic proinflammatory processes and increases cardiovascular risk [6].

The prevalence of sarcopenia in older patients with cardiovascular diseases (CVD) is high (35%), compared to 13% in the general population and varies among the different cardiovascular diagnoses (e.g., cardiac arrhythmia: 35% [7]; chronic heart failure: 32% [7] to 34% [8]; coronary artery disease: 30% [7]) [7]. The highest prevalence of sarcopenia is described between 37.5% [9] and 46.4% [10] during inpatient stay after cardiac surgery [9, 10]. The prevalence of reduced muscle mass and muscle strength in patients entering CR is high [11]. Data describing the prevalence of sarcopenia after a cardiac procedure and its impact on the patients’ functional capacity during inpatient cardiac rehabilitation (iCR) is rare.

### SARC-F questionnaire

If left untreated, sarcopenia imposes a high personal, social and economic burden. The detection of sarcopenia in cardiac patients and the optimal care of these individuals is therefore essential [12]. Especially patients with CVD requiring a cardiac procedure are at a high risk of losing skeletal muscle mass and strength, due to the periods of relative inactivity before the procedure and during convalescence.

One simple way to screen patients for sarcopenia is the Strength, Assistance with walking, Rise from a chair, Climb stairs and Falls (SARC-F) questionnaire. It is recommended by the European Working Group on Sarcopenia in Older People (EWGSOP) and aims to capture self-reports from patients on signs that are characteristic of sarcopenia [1]. The SARC-F questionnaire is a useful method in clinical practice to identify patients at a high risk for physical limitations and to predict post-discharge negative outcomes in older patients with CVD. In older patients with CVD, a SARC-F ≥ 4 is associated with higher risk of an adverse advent compared with those patients with SARC-F < 4 (adjusted hazard ratio: 1.78; 95% confidence interval: 1.03–3.07; *p* = 0.040) [13]. Furthermore, SARC-F scores ≥4 are associated with poorer results of motor function tests, quality of life as well as poorer prognosis and increased risk of rehospitalization due to CVD [14].

### Treatment of sarcopenia

The effectiveness of phase II rehabilitation in patients after a cardiovascular procedure is evident and recommended by international guidelines [15, 16]. Older and multimorbid patients in particular benefit most from iCR after cardiovascular procedure [15–20]. Older patients with sarcopenia who have had a cardiovascular procedure are likely to represent a special cohort in the setting of iCR and may have special needs within the context of therapy.

The findings of a recently published review underscore the high prevalence of age-related sarcopenia, disease-related skeletal muscle deconditioning, physical limitations, and frailty in older patients with different kinds of heart diseases [21]. The effects and safety of resistance exercise in patients with cardiac diseases have been demonstrated by numerous meta-analyses [21]. However, only few studies have addressed the feasibility and effects of resistance exercise in older physically limited and/or frail patients entering CR [21]. In a retrospective study (*n* = 322 inpatients; CVD; age 72 ± 12 years; 28% sarcopenic), Harada et al. [22] evaluated the impact of exercise training on muscle mass, muscle strength and physical performance in patients with and without sarcopenia. Sarcopenia was defined as either a gait speed of < 0.8 m/s or reduced handgrip strength (< 26 kg in males and < 18 kg in females), together with lower skeletal muscle index (SMI) (< 7.0 kg/m2 in males and < 5.7 kg/m^2^ in females). Furthermore, the actual daily total calorie and nutrient intake was calculated. The results show a significant association between SMI, protein intake and statin treatment. As a result of the exercise training, handgrip strength, gait speed, leg weight bearing index, and nutritional intake improved in patients both with and without sarcopenia [22]. The authors recommend special nutrition, physical exercise and effective medication as a useful strategy for the prevention and treatment of sarcopenia in older patients with CVD [22]. In addition, the consumption of a whey protein-based nutritional formula enriched with leucine and vitamin D improved physical performance and function, as well as muscle mass in older sarcopenic patients during their inpatient rehabilitation [23].

A cardiovascular procedure is particularly hazardous for frail older cardiac patients and can trigger an irreversible functional decline which increases the risk of care dependency [24]. Preoperative rehabilitation interventions, also termed “prehabilitation”, prior to cardiac procedures can improve perioperative outcomes and alleviate adverse effects [25–29]. Sarcopenia is one of the characteristics of frailty [30]. Especially in these patients, prehabilitation has the potential to preoperatively increase the psychological and physical ability to withstand the burdens caused by surgery [24].

In this observational pre-study to the large-scale PRECOVERY cardiac prehabilitation study [31], we aimed to assess the prevalence of sarcopenia in iCR patients ≥75 years old and quantify the functional capacity of older sarcopenic and non-sarcopenic iCR participants. The results will be used to optimize and adapt the PRECOVERY prehabilitation program to the needs of older cardiac patients requiring cardiac procedure. In order to evaluate whether and to what extent iCR is able to positively influence sarcopenia status in older CVD patients, the sarcopenia screening was repeated in the cohort after 3 months. These results were compared to the baseline data collected during iCR.

## Methods

### Patient population

Patient recruitment was carried out four iCR facilities in Germany between December 2022 and August 2023. Patients aged 75 years or older who were undergoing iCR after cardiac procedure were eligible for inclusion in the study. The eligible cardiac procedures were defined by the German operation and procedure codes catalogue (OPS) and are listed in Table 1. Patients were excluded if they were unable to understand the study information and give written consent due to poor German language skills, cognitive or visual impairments. Furthermore, patients were excluded if they were limited in their physical activity due to acute conditions (e.g., acute infection, injury).

**Table 1:** Explanation of the German operation and procedure (OPS) code

OPS Code and Procedure	
5–35: Operations on heart valves and septa as well as on vessels close to the heart	
5–351: Replacement of heart valves with prostheses	
5–352: Change of heart valve protheses	
5–353: Valvuloplasty	
5–354: Other operations on heart valves	
5-35a: Minimally invasive surgery on heart valves	
5–36: Coronary artery surgery	
5–360: Desobliteration (endarterectomy) of coronary arteries	
5–361: Creating an aortocoronary bypass	
5–362: Creating an aortocoronary bypass using minimally invasive technique	
5–363: Other revascularisation of the heart	

Abbreviations. *OPS* operation and procedure codes

### Study setting

This is an observational cohort pre-study of the ongoing randomized, controlled, two-arm parallel group, assessor-blinded multicenter prehabilitation study “PRECOVERY” [31]. In Germany, CR is offered as an inpatient or outpatient measure. The duration varies between 3 - 4 weeks, depending upon the patients´ condition [32].

The main tasks of CR are individual goal setting, cardiovascular prevention, and psychosocial interventions [15]. Physical activity and exercise training build the cornerstones of contemporary CR programs resulting in the worldwide accepted term of “exercise-based cardiac rehabilitation” [16].

**Fig. 1:**
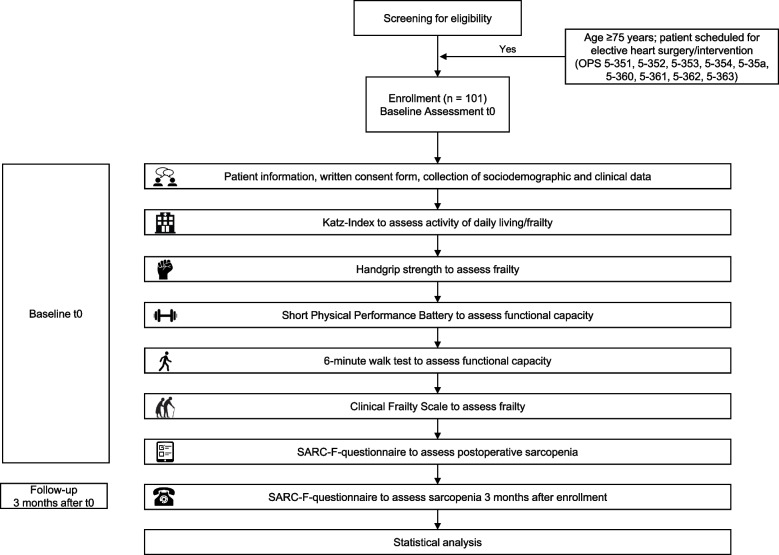
Cohort study flow chart. Abbreviations: *SARC-F* Strength, Assistance with walking, Rise from a chair, Climb stairs and Falls

Eligible patients were asked to participate in the cohort study by physicians at the iCR facilities during regular clinical consultations. After recruitment, all participants were informed about the study goals, duration of the study, the role of each participant, and any risks in written as well as oral forms by the study coordinators. All patients provided written informed consent. Subsequently, the baseline assessment took place (details see Fig. 1).

#### Sample size calculation

We calculated the power of a pre-post comparison of the SARC-F score measured at baseline and 3 months after the baseline assessment. Assuming a mean difference in scores of θ = 1 and a standard deviation of σ = 2, e.g. an effect size Δ = θ/σ = 0.5, a one-sided Wilcoxon signed-rank test with *n* = 46 pairs (pre-post) will have a power of 90% to detect such an effect when testing to the significance level of α = 2.5%. With *n* = 36 the same test would still have a power of 80% to detect such an effect. Sample size calculations were performed using nQuery 9.2.1.0 (GraphPad Software DBA Statistical Solutions, USA).

### Sociodemographic data and medical history

The sociodemographic data includes age, gender, the level of education, main profession, housing situation and the level of care. The medical history consists of the parameter number of medications (regularly and pro re nata), cardiovascular indications for the cardiac rehabilitation stay as well as cardiac and non-cardiac concomitant diseases.

### Functional capacity and frailty outcomes

Functional capacity and frailty outcomes were the results of the Katz-Index, the CFS, the handgrip strength (HGS), the Short Physical Performance Battery (SPPB) and the 6-minute walk test (MWT) measured during the baseline assessment at iCR. Additionally, the SARC-F score was measured at baseline and 3 months after the baseline assessment.

The main research questions are:

- Does the participation in an iCR after cardiac procedure lead to a significant improvement in sarcopenia status measured by SARC-F questionnaire 3 months after the baseline assessment?
- How prevalent is sarcopenia in the cohort at iCR, measured with the SARC-F questionnaire?
- Is there a significant correlation between sarcopenia measured by the SARC-F questionnaire and functional capacity (measured with HGS, SPPB score and 6MWD) at baseline?

Table 2 shows the range of values and the cut-off values of the current literature of the assessments used at baseline [1, 2, 33–35].

**Table 2:** Range of values and cut-off values of the baseline assessments for pathological findings

	Range of values	Cut-off values for pathological finding
Katz-Index	0–6	Score < 6 [33]
Handgrip strength	N/A	♂ ≤ 27 kg; ♀ ≤ 16 kg [1]
SPPB score	0–12	Points ≤ 8 [1]
6MWD (m)	N/A	< 300 m [34]
CFS	1–9	≥ 4 [35, 36]
SARC-F	0–10	≥ 4 [2]

Abbreviations: *SPPB* Short Physical Performance Battery, *m* meter, *CFS* Clinical Frailty Scale, *SARC-F* Strength, Assistance with walking, Rise from a chair, Climb stairs and Falls, *N/A* not available

#### Sarcopenia assessment (SARC-F)

The SARC-F questionnaire is a validated assessment tool for the diagnosis of sarcopenia. It is recommended by the EWGSOP to identify individuals at risk for sarcopenia [1, 37]. It consists of five questions assessing muscle strength, gait disturbance, and falls. A maximum score of 10 points can be obtained. A score ≥ 4 suggests sarcopenia [38, 39]. The questionnaire has an excellent specificity (85%) with a negative predictive value of 96%. However, it also has a low sensitivity (75%) and a positive predictive value of 42% [38–40].

#### The Katz-index

To assess patients´ daily activity the Katz-Index was used. The Katz-Index [41] provides valid information about how (in)dependent a patient is in the performance of activities of daily living. The index consists of the following six items: bathing/washing, dressing, toileting, transfer, continence, and feeding [41].

#### The clinical frailty scale

Frailty was assessed using the CFS. The CFS emerged from the Canadian Study of Health and Aging (CSHA). The German translation of the CFS consists of nine categories, whereby a CFS of 1 defines a very fit person and a CFS of 9 a terminally ill person. Frailty is defined as a score of 4 or higher on the CFS [35, 36].

#### Assessments of functional capacity (HGS, SPPB, 6MWT)

HGS was assessed in all participants using a hand dynamometer (Jamar Hand Dynamometer, IL, USA) [42]. The measurement is carried out according to the Clinical Assessment Recommendations of the American Society of Hand Therapists (ASHT) [43]. Participants were asked to start the HGS assessment with their dominant hand (right-handed or left-handed) followed by the non-dominant hand. Then, the test was repeated with the stronger hand. The second attempt with the stronger hand was defined as the maximal HGS.

The SPPB is a test battery for the measurement of motor function of the lower limbs [44]. It is a reliable and valid measurement instrument that is used primarily in geriatric patients to assess their mobility [45]. The test battery consists of a balance test, 4-m walk and sit-to-stand test. A maximum of 12 points can be achieved, which provide information about the patients´ impairment of daily living (0–3 points: severe impairment; 4–6 points: moderate impairment; 7–9 points: mild impairment; 10–12 points: no everyday life impairment) [45].

The 6MWT is an easy-to-perform test that does not require any additional equipment or preparatory training of the test persons. The test is performed according to the recommendations of the American Thoracic Society [46] and the distance covered in 6 min was recorded in meters. A 6MWD < 300 m is associated with high mortality risk and/or poor health status as well as reduced results in physical function tests (e.g., HGS, one-leg standing time) [34, 47].

### Statistical analysis

We used the Kolmogorov–Smirnov test for the assessment of normal distributions. Continuous and categorical variables are presented by mean ± standard deviation with absolute and relative frequencies, respectively. Two-group comparisons of baseline variables were performed using Students´ t-test and Chi-square-test of independence for continuous and categorical variables. To analyze the results of the pre-post-test, the Wilcoxon signed-rank test as a non-parametric test for two dependent samples was applied. Effect size was calculated by $r=\frac{z}{\surd n}$. The interpretation values for the effect size are: 0.10 ≤ r < 0.3 (small effect), ≤ 0.30 r < 0.5 (moderate effect) and r > 0.5 (large effect) [48]. For the calculation of the bivariate correlation between the nominal variable sarcopenia and the interval variables HGS, SPPB and 6MWD, the eta coefficient with one-way analysis of variance (ANOVA) was conducted. In all analyses, a *p* < 0.05 was considered statistically significant. Analyses were performed using IBM Statistical Package for the Social Sciences (SPSS) Version 21.0 (IBM Co., Armonk, NY, USA).

## Results

### Study population

**Fig. 2:**
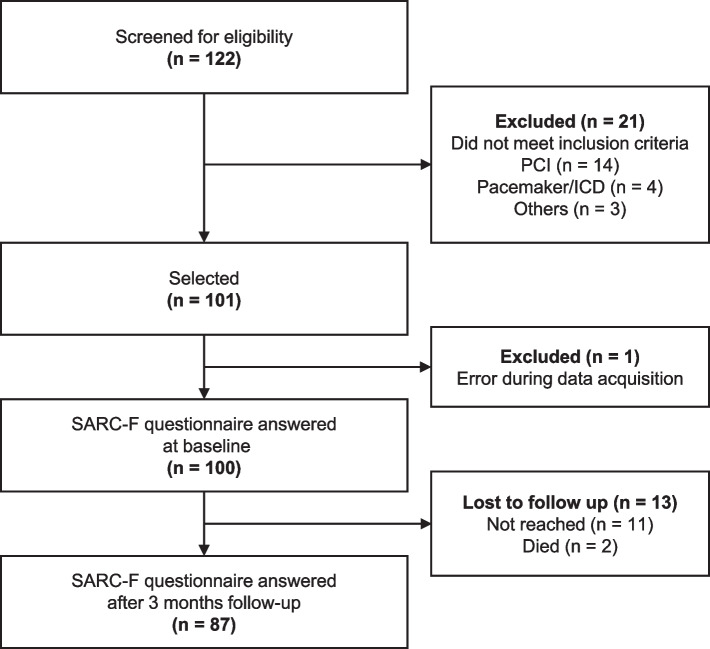
Flow diagram of the convenient sample. Abbreviations: *PCI* Percutaneous Coronary Intervention, *ICD* Implantable cardioverter-defibrillator, *SARC-F* Strength, Assistance with walking, Rise from a chair, Climb stairs and Falls questionnaire

A total of 122 patients were screened for eligibility. Of these, 21 were excluded due to ineligible diagnoses. A sample of 101 patients (79.7 ± 4.0 years; 63% males) were included into the study. Of the total cohort, 87 participated in the follow-up screening (drop-out rate: 14%). Of the 13 dropouts, two patients died and 11 could not be reached by telephone (Fig. 2). The drop-out analysis showed that patients lost to follow-up were significantly older (*p* = 0.013), more likely to live alone (*p* = 0.001) and had significantly more often chronic pain than patients who participated in the three-month follow-up. All other variables did not differ between the groups.

Table 3 presents the patient characteristics at baseline. One patient of the cohort did not complete the SARC-F questionnaire at baseline due to technical reasons, therefore he could not be assigned to one of the groups. The SARC-F questionnaire was used to identify sarcopenic patients. Patients with a score ≥ 4 points were considered to be sarcopenic. Based on the SARC-F score, the participants were divided into sarcopenic (*n* = 35) and non-sarcopenic patients (*n* = 65). At baseline, 35% of the cohort was diagnosed to be sarcopenic (SARC-F score of ≥4 points). Three months after iCR, the number of patients with a SARC-F score of ≥4 points had decreased to 23 (23%).

**Table 3:** Patient characteristics

Characteristics	All	sarcopenic patients	non-sarcopenic patients	*P*-value
	(*n* = 101)	(*n* = 35)	(*n* = 65)	
	*(mean ± SD) or*	*(mean ± SD) or*	*(mean ± SD) or*	
	*(n, %)*	*(n, %)*	*(n, %)*	
Age (years)	79.7 ± 4.0	82.1 ± 4.5	78.4 ± 3.1	* < 0.001
Gender				
male	64 (63%)	14 (40%)	49 (75%)	^**#**^ **< 0.001**
female	37 (37%)	21 (60%)	16 (25%)	^**#**^ **< 0.001**
Number of medications (regularly)	9.0 ± 3.0	10.1 ± 3.4	8.5 ± 2.6	*0.009
Number of medications (p.r.n.)	1.3 ± 1.4	1.1 ± 1.3	1.4 ± 1.4	*0.231
Level of care	24 (24%)	16 (46%)	8 (12%)	^**#**^**0.001**
Situation of living				
Living alone	27 (27%)	17 (49%)	10 (15%)	^**#**^ **< 0.001**
Diagnosis^a^				
CABG-surgery	44 (33%)	7 (18%)	36 (39%)	^**#**^**0.001**
Valve surgery (e.g., mitral valve replacement)	50 (38%)	16 (41%)	34 (37%)	^#^0.373
Valve intervention (e.g., MitraClip, TAVI)	20 (15%)	13 (33%)	7 (8%)	^**#**^**0.002**
Concomitant diseases				
Coronary artery disease	73 (72%)	23 (66%)	49 (75%)	^#^0.304
Myocard infarction	21 (21%)	5 (14%)	16 (25%)	^#^0.226
Heart failure	75 (74%)	28 (80%)	46 (71%)	^#^0.315
Cardiac arrhythmias	64 (63%)	25 (71%)	38 (59%)	^#^0.200
Stroke	10 (10%)	4 (11%)	6 (9%)	^#^0.727
Peripheral artery disease	12 (12%)		6 (9%)	^#^0.246
Hypertension	92 (91%)	31 (89%)	60 (92%)	^#^0.533
Diabetes mellitus	27 (27%)	8 (23%)	16 (25%)	^#^0.844
Asthma bronchiale	10 (10%)	6 (17%)	4 (6%)	^#^0.081
Chronic lung disease (e.g. COPD)	13 (13%)	6 (17%)	7 (11%)	^#^0.366
Rheumatism	6 (6%)	2 (6%)	4 (6%)	^#^0.930
Athrosis	34 (34%)	18 (51%)	16 (25%)	^**#**^**0.007**
Gout	15 (15%)	8 (23%)	7 (11%)	^#^0.106
Fractures	51 (51%)	21 (60%)	30 (46%)	^#^0.186
Chronic pain	16 (16%)	10 (29%)	5 (8%)	^**#**^**0.005**
Kidney disease	27 (27%)	13 (37%)	14 (22%)	^#^0.076
Cancer				
Breast	6 (6%)	2 (6%)	4 (6%)	^#^0.930
Prostate	12 (12%)	2 (6%)	10 (15%)	^#^0.156
Colon	3 (3%)	2 (6%)	1 (2%)	^#^0.243
Baseline Assessments				
KATZ-Index (PPF)	**5.7 ± 0.9** (17.8%)	**5.1 ± 1.3** (38.9%)	**5.9 ± 0.3** (6.2%)	*0.005^**†**^
Handgrip strength (kg, PPF)	**24.9 ± 9.9**	**17.5 ± 8.3**	**28.7 ± 8.3**	* < 0.001^**†**^
	(♂: 37.5%; ♀: 56.8%)	(♂: 60.0%; ♀: 71.4%)	(♂: 30.6%; ♀: 37.5%)	
SPPB score (PPF)	**7.5 ± 3.3** (57.4%)	**4.7 ± 2.8** (94.4%)	**9.0 ± 2.5** (36.9%)	* < 0.001^**†**^
6MWD (m, PPF)	**288.8 ± 136.5** (43.6%)	**186.5 ± 120.7** (75.0%)	**342.6 ± 112.4** (26.2%)	* < 0.001^**†**^
CFS (PPF)	**3.2 ± 1.4** (41.6%)	**4.1 ± 1.0** (66.6%)	**2.7 ± 1.3** (27.6%)	* < 0.001^**†**^

Abbreviations: *SD* standard deviation, *n* number, e.g. for example, *TAVI* transcatheter aortic valve implantation, *COPD* chronic obstructive pulmonary disease, *p.r.n.* Pro re nata [as needed], *PPF* percentage of pathological findings, * Independent t-test, *#* Chi-square-test, ^a^Multiple responses, *†* Bonferoni correction

Notably, 71% of female and 60% of male patients in the sarcopenic group did not reach the cut-off values from the EWGSOP in HGS. The same trend was seen in SPPB score where 94% of the sarcopenic and 37% of the non-sarcopenic participants did not reach the cut-off values [1] (see Table 3).

The group of sarcopenic patients were significant older (*p* < 0.001), predominantly female (*p* < 0.001), lived mainly alone (*p* < 0.001) and were more likely to receive some degree of nursing assistance (*p* = 0.001) than non-sarcopenic patients. Patients with a SARC-F score ≥ 4 points had a higher prevalence of arthrosis (*p* = 0.007) and chronic pain (*p* = 0.005) in comparison to participants with SARC-F scores ≤3 points. Patients classified as sarcopenic were more likely to receive iCR due to heart valve surgery (*p* = 0.002) and less likely to have had CABG surgery (*p* = 0.001), compared to non-sarcopenic patients. A gender-specific analysis of the assessment results can be found in the Additional file 1.

### Outcomes

The results of the Katz-Index, CFS, HGS, SPPB and 6MWT at baseline are summarized in Table 3. It is important to note that these results show that sarcopenic patients performed significantly worse in the assessments compared to non-sarcopenic patients.

**Fig. 3:**
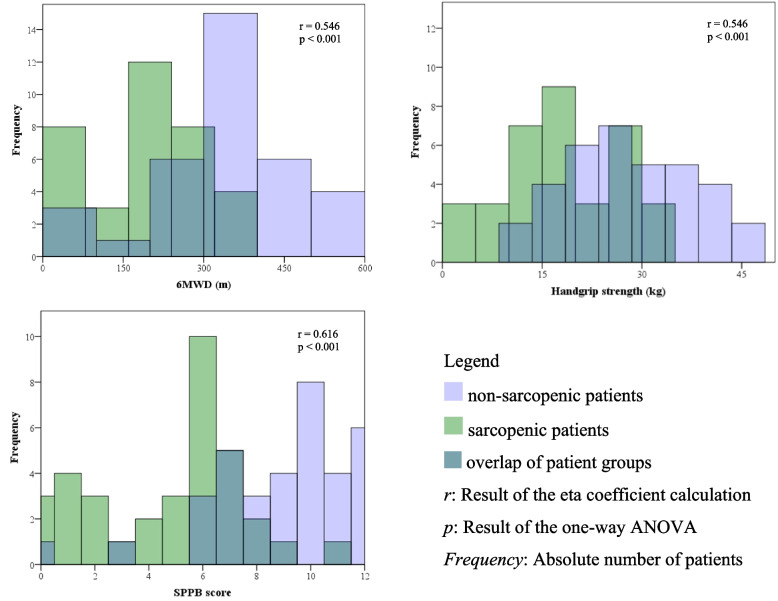
Overlapping histograms illustrating the correlation between sarcopenia and 6MWD, HGS and SPPB score. Abbreviations: *6MWD* 6-minute walk distance, *HGS* handgrip strength, *SPPB* Short Physical Performance Battery

The calculation of the eta coefficient with one-way ANOVA revealed a significant correlation between sarcopenia measured by the SARC-F questionnaire and the 6MWD (r = 0.546; *p* < 0.001), SPPB score (r = 0.616; *p* < 0.001) and HGS (*r* = 0.546; *p* < 0.001). Figure 3 illustrates the overlapping histograms of each correlation.

**Fig. 4:**
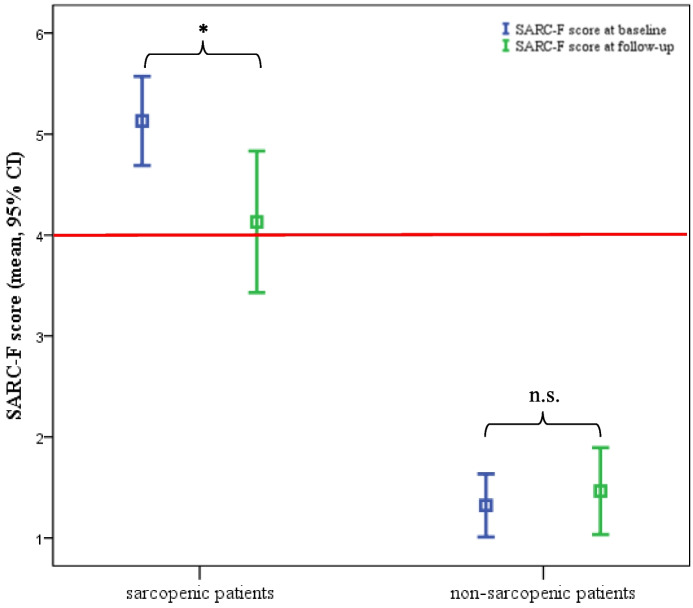
SARC-F score results of the pre-post-test for sarcopenic and non-sarcopenic patients. Abbreviations: * significant changes, *p* = 0.017, z = − 2387, r = 0.43, *n.s.* not significant, *p* = 0.739, *red line* cut-off value for SARC-F score which is a mark for sarcopenia, *SARC-F* Strength, Assistance with walking, Rise from a chair, Climb stairs and Falls questionnaire

The Wilcoxon-Test showed no significant development in the total sample in the SARC-F score 3 months after iCR (2.7 ± 2.1 vs. 2.4 ± 2.1, *p* = 0.207) but a significant decrease in patients with post-procedural sarcopenia (5.1 ± 1.2 vs. 4.1 ± 1.9, *p* = 0.017) (see Fig. 4). In the sub-group of patients with post-procedural sarcopenia (*n* = 31), 18 improved (58%), 7 showed poorer results (23%) and 6 did not change (19%) in SARC-F score between baseline and 3 months follow-up (lost to follow-up: *n* = 4). In the non-sarcopenic group (*n* = 56), 17 (30%) improved, 15 (27%) showed poorer results, and 24 (53%) did not change their in SARC-F score in the pre-post-test.

## Discussion

In this observational cohort study, we evaluated the prevalence of sarcopenia and quantified the functional capacity of older patients at iCR after cardiac procedure. This is the first study which has collected SARC-F scores 3 months after iCR and compared these findings with the functional baseline assessments performed during the iCR stay. Likewise, our cohort consists only of cardiac patients aged 75 years and older after cardiac surgery. The data were collected in four different iCR facilities, which has a generalizable value. All other studies in this field have collected data monocentrically in younger cohorts with different iCR diagnosis.

Approximately every third patient in the cohort exceeded the threshold for sarcopenia. Sarcopenic patients performed significantly worse in all functional tests in comparison to non-sarcopenic patients. In addition, we screened for sarcopenia 3 months after iCR and compared these results to the data collected at baseline, finding a slightly lower prevalence of sarcopenia (about 1 in 5 participants) at follow-up.

### Sarcopenia assessment

The results demonstrate 35% of the cohort to be sarcopenic (SARC-F score ≥ 4 points) at iCR. In comparison to non-sarcopenic patients, sarcopenic patients were mostly older, more likely to live alone, be female, take more regular medications, have undergone a valve intervention and receive some degree of nursing assistance. These findings are in line with those from Harada et al. [22] and Tanaka et al. [13]. Both authors described a SARC-F score ≥ 4 in 26.8% [13] to 28.0% [22] of their patients cohort. Likewise, patients with sarcopenia were significant older [13, 22], female [22] and had more comorbidities [13] than non-sarcopenic patients.

The SARC-F questionnaire is a useful screening tool for assessing impaired physical function in older CVD patients but its use in the clinical setting is rare. Tanaka et al. [13] used the SARC-F questionnaire to assess sarcopenia in CVD patients 65 years and older before hospital discharge. The sarcopenic group assessed by Tanaka et al. [13] (SARC-F score ≥ 4) had significantly lower handgrip strength, leg strength, and respiratory muscle strength, poorer standing balance, slower than usual gait speed, lower SPPB score, and shorter 6-minute walking distance compared to the non-sarcopenic group [13]. Compared to our results, the cohort evaluated by Tanaka et al. [13] scored lower (i.e. less prevalence of sarcopenia) in the SARC-F. This could be due to the younger age of the cohort evaluated (75.8 ± 6.7 vs. 79.7 ± 4.0 years). Furthermore, only 35.3% of the cohort had a CABG surgery whereas in our cohort, a cardiac procedure was one of the inclusion criteria [13]. In a cohort with a similar mean age compared to our cohort, Noda et al. [14] used the SARC-F questionnaire to detect sarcopenia in older CVD patients with cognitive impairment. The results revealed similar SARC-F scores in the main cohort as well as in the sarcopenic and non-sarcopenic groups [14].

In the follow-up 3 months after iCR, results showed that 23% of the cohort was classified as sarcopenic. In patients with post-procedural sarcopenia, we observed a significant decrease in SARC-F score 3 months after iCR, indicating less sarcopenia. However, no changes were observed in the total cohort. These results indicate that especially sarcopenic patients with CVD benefit in this respect from a multimodal iCR. This assumption is supported by the findings of Harada et al. [22] which show significant improvements in gait speed, muscle strength, including handgrip and leg weight bearing index, Barthel index and peak VO2/Watt as evidence of the success of iCR [22].

### Assessments of functional capacity

The results of our cohort study revealed sarcopenic patients score significantly poorer in assessments evaluating the performance of activities of daily living, frailty as well as functional capacity, compared to non-sarcopenic patients. To identify limitations in muscle strength and physical performance, the EWGSOP described cut-off values for HGS and SPPB score [1]. HGS is an indicator of overall strength, provides insight into physical function and prognosis [49]. A low HGS is a predictor of poor patient outcomes such as longer hospital stays, increased functional limitations, poor health-related quality of life and death [50, 51]. HGS correlates significantly with gender, height, peakVO2 and age [42]. Patients with coronary artery disease participating in iCR have been demonstrated to improve HGS significantly by 4.6% [49]. In our cohort, the mean HGS was lower in sarcopenic patients than in the non-sarcopenic patients. These findings are in line with the findings reported by Yuenyongchaiwat et al. [10] and Harada et al. [22]. HGS values assessed at iCR vary in these cohorts from 14.2 kg [22] to 17.5 kg [10] in sarcopenic and 27.3 kg [10] to 27.6 kg [22] in non-sarcopenic patients. Additional analysis in our cohort study showed that 71% of females and 60% of males in the sarcopenic group did not meet the cut-off values, indicating that a large post-procedural deficit in muscle strength is to be expected in sarcopenic patients after cardiac procedure. Consequently, cardiac prehabilitation programs should include specific muscle strengthening exercises to counteract these deficits, especially in patients with sarcopenia.

The SPPB is a well-established tool to assess physical performance and to identify frail patients [1, 52]. Furthermore, it is useful to guide the management of older frail patients in the post-acute phase after a cardiac procedure during iCR [52]. The SPPB score is significantly associated with sarcopenia in older cardiac patients and a cut-off point is described by a score from 9/10 [53]. Rinaldo et al. [52] used the SPPB for assessment in a group of older patients after cardiac event (70.9% after cardiac surgery) at entry and discharge to iCR (average length of stay 22.2 ± 10.4 days). As a result of the iCR, the SPPB score improved significantly (6.9 ± 3.1 to 8.4 ± 3.5) whereas the improvements were more pronounced in patients with severe/moderate limitations than in in those with mild or minimal/no limitations [52]. Rengo et al. [54] used the SPPB to assesses physical function in patients ≥65 years (26% after CABG surgery) in a similar setting. As a result of the iCR, the SPPB score improved significantly (9.9 ± 0.2 vs. 10.7 ± 0.2) [54]. In our cohort study, we detected significantly lower SPPB scores in the sarcopenic patients than in the non-sarcopenic group; 94% of the sarcopenic and 37% of the non-sarcopenic participants in our cohort study did not reach the cut-off values from the EWGSOP. Comparable results are also reported by Yasuda et al. [53]. These findings emphasize the importance of specific balance and coordination exercises in addition to resistance exercise as a part of cardiac prehabilitation programs to counteract physical limitation due to deficits in the motor function of the lower limbs, especially in patients with sarcopenia.

Functional capacity measured by 6MWT is an important prognostic predictor for future cardiac events and mortality [55] and can also be used as an indicator of recovery in mobility after cardiac surgery [56]. The average 6MWD after cardiac procedure at hospital discharge/beginning of iCR described in other studies varies between 179.1 ± 92.2 m and 331.6 ± 107.9 m [56–59]. The 6MWD correlates significantly by age, gender, regular exercise, comorbidity, left ejection fraction and preoperative New York Heart Association (NYHA) classification [57, 58]. In our cohort study, 6MWD in patients with sarcopenia was significantly shorter (186.5 ± 120.7 m; *p* < 0.001) than in the non-sarcopenic patients (342.6 ± 112.4 m). Both results are in line with the results found in the current literature [56–59]. A recently published randomized control trial showed that a prehabilitation program before elective cardiac surgery improved 6MWD significantly in the subgroup of sarcopenic patients [60]. These results indicate that cardiac prehabilitation programs should include exercises to increase endurance and mobility to prepare especially older patients with sarcopenia for the procedure and the convalescent phase of relative inactivity.

### Perioperative optimal treatment

The results described above show that there is a large deficit in functional capacity in older CVD patients during their perioperative pathway from hospital admission until iCR discharge. To counteract post-procedural sarcopenia in this cohort, a preoperative screening of participants with the SARC-F questionnaire could be initiated in the clinical setting once patients are placed on the waiting list for an elective cardiac procedure. Active regular screening for sarcopenia could be effective in detecting high risk patients and offer them multimodal perioperative care. Especially in older patients with sarcopenia, prehabilitation should include not only exercise training (e.g., strength training) but also modules to optimize nutrition, improve iron deficiency and anemic status to improve patients´ post-procedural outcome.

### Limitations

This is a relatively small cohort study. Further studies with larger cohorts are needed to confirm the presented results. A limitation of this prospective cohort study is the missing control group. This reduces the significance of the results. Further studies with randomized controlled design are needed to confirm the results. Likewise, instead of a 3 months follow-up by telephone, a re-survey in the clinical setting with a repeat of all baseline assessments would be desirable. Based on this, more accurate conclusions could be drawn about the development of functional capacity in sarcopenic patients and the need for both prehabilitation and additional, maintenance treatment following iCR discharge.

## Conclusions

The incidence of sarcopenia in older patients entering iCR after a cardiac procedure is high (35%) and remains high at 3 months follow-up (23%). In the total sample, no significant changes in the SARC-F score at 3 months follow-up were observed, whereas the sub-group of patients with post-procedural sarcopenia improved significantly. The diagnosis of sarcopenia in these patients correlates with poor functional capacity. These results indicate that this group of patients could benefit from prehabilitation for physical and psychological stabilization prior to cardiac procedure, thereby improving perioperative outcomes, increasing functional capacity and mitigating adverse effects.

### Supplementary Information

**Additional file 1.** Gender-specific analysis of the assessment results

## Data Availability

The dataset will be available from the corresponding author on reasonable request.

## Abbreviations

ASHT: American Society of Hand Therapists
CABG: Coronary artery bypass graft surgery
CFS: Clinical Frailty Scale
CR: Cardiac rehabilitation
CSHA: Canadian Study of Health and Aging
CVD: Cardiovascular diseases
DRKS: German Clinical Trials Register [Deutsches Register Klinischer Studien]
e.g.: For example [exempli gratia]
EWGSOP: European Working Group on Sarcopenia in Older People
HGS: Handgrip strength
iCR: inpatient cardiac rehabilitation
m: meter
NYHA: New York Heart Association
OPS: Operation and procedure codes catalogue
SARC-F: The Strength, Assistance with walking, Rise from a chair, Climb stairs and Falls
SMI: Skeletal muscle index
SPPB: Short Physical Performance Battery
WHO: World Health Organization
6MWD: 6-minute walk distance
6MWT: 6-minute walk test
