# Supplementary material for: The prevalence and impact of sarcopenia in older cardiac patients undergoing inpatient cardiac rehabilitation – results from a prospective, observational cohort pre-study
Source: BMC Geriatr. 2024 Jan 24;24:94. doi: 10.1186/s12877-024-04694-y (PMC10809534; doi:10.1186/s12877-024-04694-y)
Supplement: Supplementary file 1 — Additional file 1. Gender-specific analysis of the assessment results [file 12877_2024_4694_MOESM1_ESM.pdf]

Additional file 1 Gender-specific analysis of the assessment results

Characeristics	All (n = 101)<br>Female (n = 37)<br>Male (n = 64)<br>*(mean ± SD)*	sarcopenic patients (n = 35)<br>Female (n = 21)<br>Male (n = 14)<br>*(mean ± SD)*	non-sarcopenic patients (n = 65)<br>Female (n = 16)<br>Male (n = 49)<br>*(mean ± SD)*	P-value
KATZ-Index				
All	5.7±0.9	5.1±1.3	5.9±0.3	***p=0.001**
Female	5.6±1.0	5.2±1.2	6.0±0.0	***p=0.010**
Male	5.7±0.8	5.0±1.5	5.9±0.4	***p=0.040**
Handgrip strength (kg)				
All	24.9±9.9	17.5±8.3	28.7±8.3	***p<0.001**
Female	15.5±6.8	12.3±5.7	19.8±5.9	***p<0.001**
Male	30.3±6.8	25.2±4.6	31.6±6.8	***p=0.002**
SPPB score				
All	7.5±3.3	4.7±2.8	9.0±2.5	***p<0.001**
Female	6.3±3.3	5.0±2.8	8.1±3.2	***p=0.003**
Male	8.2±3.1	4.5±3.0	9.3±2.2	***p<0.001**
6MWD (m)				
All	288.8±136.5	186.5±120.7	342.6±112.4	***p<0.001**
Female	215.3±124.2	184.7±108.8	255.4±134.9	*p=0.086
Male	331.2±125.6	189.3±141.1	371.0±88.3	***p<0.001**
CFS				
All	3.2±1.4	4.1±1.0	2.7±1.3	***p<0.001**
Female	3.7±1.2	4.1±1.1	3.1±1.2	***p=0.009**
Male	2.9±1.4	4.1±1.0	2.6±1.3	***p<0.001**

Abbreviations: *n* number, *SD* standard deviation, *kg* kilogram, *SPPB* Short Physical Performance Battery, *6MWD* 6-minute walk distance, *m* meters, *CFS* Clinical Frailty Scale.
